# T Helper Lymphocyte and Mast Cell Immunohistochemical Pattern in Nonceliac Gluten Sensitivity

**DOI:** 10.1155/2017/5023680

**Published:** 2017-12-07

**Authors:** Giuseppe Losurdo, Domenico Piscitelli, Federica Pezzuto, Francesco Fortarezza, Claudia Covelli, Antonella Marra, Andrea Iannone, Annacinzia Amoruso, Mariabeatrice Principi, Enzo Ierardi, Alfredo Di Leo

**Affiliations:** ^1^Section of Gastroenterology, Department of Emergency and Organ Transplantation, University “Aldo Moro”, Bari, Italy; ^2^Section of Pathology, Department of Emergency and Organ Transplantation, University “Aldo Moro”, Bari, Italy

## Abstract

**Background and Aims:**

Nonceliac gluten sensitivity (NCGS) is a gluten-related emerging condition. Since few data about NCGS histopathology is available, we assessed the markers of lymphocyte and innate immunity activation.

**Materials and Methods:**

We retrieved duodenal biopsy samples of patients with NCGS diagnosis according to the Salerno criteria. We selected specimens of positive (seropositive celiac disease/Marsh 1-2 stage) and negative (normal microscopic picture) controls. Immunohistochemistry for CD3 (intraepithelial lymphocytes-IELs), CD4 (T helper lymphocytes), CD8 (T cytotoxic lymphocytes), and CD1a/CD117 (Langerhans/mast cells) was performed. ANOVA plus Bonferroni's tests were used for statistical analysis.

**Results:**

Twenty NCGS, 16 celiac disease, and 16 negative controls were selected. CD3 in NCGS were higher than negative controls and lower than celiac disease (18.5 ± 6.4, 11.9 ± 2.8, and 40.8 ± 8.1 IELs/100 enterocytes; *p* < 0.001). CD4 were lower in NCGS than controls and celiac disease (31.0 ± 22.1, 72.5 ± 29.5, and 103.7 ± 15.7 cells/mm^2^; *p* < 0.001). CD8 in NCGS were similar to negative controls, but lower than celiac disease (14.0 ± 7.4 and 34.0 ± 7.1 IELs/100 enterocytes, *p* < 0.001). CD117 were higher in NCGS than celiac disease and negative controls (145.8 ± 49.9, 121.3 ± 13.1, and 113.5 ± 23.4 cells/mm^2^; *p* = 0.009).

**Conclusions:**

The combination of CD4 and CD117, as well as IEL characterization, may be useful to support a clinical diagnosis of NCGS.

## 1. Introduction

Nonceliac gluten sensitivity (NCGS) is a clinical condition characterized by intestinal and extraintestinal symptoms occurring after gluten ingestion in subjects who are neither affected by celiac disease nor by wheat allergy [1].

The clinical presentation is a combination of intestinal symptoms (abdominal pain, bloating, and diarrhea) and extraintestinal manifestations (headache, asthenia, depression, joint, and muscular pain) [2]. The latency time between the ingestion of gluten and the onset of symptoms is short, within hours or days, and furthermore, a prompt symptom improvement is experienced with a gluten-free diet [3]. The prevalence of such condition seems to be more common in women and young people even if it is unknown in the general population [1]. This is due to the lack of an objective diagnostic tool, which may guide appropriate studies, as well as the heterogeneous circumstances affecting the decision to start a gluten-free diet (automedication, media or internet suggestions, empirical proposal of dieters, or nutritionists without a medical advice).

From a pathophysiological point of view, while both the adaptive and innate immune systems are involved in celiac disease, in NCGS, an isolate dysfunction of the innate immune system is hypothesized to occur. For instance, patients with NCGS show increased levels of Toll-like receptors- (TLR-) 2 and toll-like receptors- (TLR-) 4, which are receptors activated by nonself-antigens during innate immune response and overexpression of alpha and beta intraepithelial lymphocytes (IELs) [4]. On the other hand, Sapone et al. demonstrated that there are no differences in the expression of adaptive immunity markers, such as interleukin-6, interleukin-21, and interferon-gamma. Therefore, a prevalent involvement of innate immunity could explain the clinical, serological, and histological differences between NCGS and celiac disease [5].

NCGS shows a peculiar serologic pattern. Different from celiac disease, antitransglutaminase and antiendomysium antibodies are negative, while 50% of the patients have antigliadin native antibody positivity [6]. Different from celiac disease, human leukocyte antigen (HLA) DQ2-DQ8 genotypes have been observed only in 50% of the patients affected by NCGS. In the absence of sensitive and specific diagnostic tests as well as well-defined clinical features, the diagnosis of NCGS remains a diagnosis of exclusion based on subjective criteria [7].

Furthermore, patients with NCGS show a mild-to-moderate mucosal inflammation, characterized by increased IEL infiltration, with a variable microscopic picture ranging from a normal morphological status (Marsh classification grade 0) to mild alterations (Marsh grade 1), such as intraepithelial lymphocytosis (>25 IELs/100 enterocytes) with irrelevant changes in villous architecture [8, 9]. Such histological pattern is shared by many other emerging conditions, that is, microscopic enteritis. This new entity, characterized by an abnormal infiltration of IELs in intestinal epithelium, appears to be linked to either gluten- or nongluten-related diseases [10, 11].

A recent article suggests that linear T-lymphocyte infiltration in the lamina propria may be a marker of NCGS. However, this finding has been observed in a single-center experience in about 78.5% of the patients [12]. Eosinophilia in the lamina propria has also been proposed as another possible characteristic feature [2]. Despite these sporadic reports, a typical histological hallmark of NCGS has not been found so far. Therefore, in the present study, we aimed to identify specific immunohistochemical features of NCGS by investigating the pattern of the markers of lymphocyte and innate immunity activation.

## 2. Methods

### 2.1. Patients

The present study was retrospectively designed to investigate immunohistochemical features of the duodenum of patients with a previous diagnosis of NCGS.

Consecutive patients referred to our gastroenterology unit in the period June 2012–June 2013 with NCGS identified according to Salerno criteria [13] were retrospectively selected. In detail, all patients underwent a double-blind crossover strategy in order to ensure a reliable diagnosis of NCGS. Moreover, all possible causes of microscopic enteritis were excluded according to Bucharest consensus conference [10] and based on a previous experience of our group [9]. All patients underwent serology for celiac disease (antitransglutaminase IgA and, where required, IgG, and antiendomysium) and upper endoscopy with duodenal biopsy. Wheat allergy was ruled out as well by both prick test for wheat and specific radioallergosorbent test for gliadin. Other alimentary allergies have been recorded based on patient clinical history.

Biopsy samples of NCGS had been taken from patients under gluten-containing diet. We excluded subjects aged less than 18 years or unwilling to participate to the study. Duodenal samples from positive control group with serologically positive celiac disease at Marsh 1 or 2 stage before starting a gluten-free diet were retrieved, according to the guidelines for celiac disease diagnosis [14]. Patients with a normal histological picture of the duodenum, in which possible causes of microscopic enteritis had been excluded, represented the negative control group [10]. Positive and negative controls were consecutively enrolled in the same center and period.

All the patients had given a written consent for endoscopic procedure and agreed to the manipulation of their biopsy samples for histological reassessment and immunohistochemistry. During the endoscopic procedure, at least four biopsies had been taken in the duodenum and fixed in 10% formalin, embedded in paraffin, and analyzed by immunohistochemistry.

### 2.2. Immunohistochemistry

Collection and processing were performed according to biospecimen reporting for improved study quality (BRISQ) recommendations [15]. Immunohistochemical analysis of CD3, CD4, CD8, CD1a, and CD117 was performed.

All primary antibodies used for immunohistochemistry were monoclonal and obtained as follows:

1. CD3 was purchased from Novocastra (Menarini, Milano, Italy) and, after a pretreatment with EDTA, used at a 1 : 100 dilution.
2. CD4 was retrieved from DAKO (Glostrup, Denmark) and used with EDTA pretreatment at 1 : 50 dilution.
3. CD8 was purchased from Novocastra (Menarini, Milano, Italy) and, after a pretreatment with EDTA, used with a 1 : 50 dilution.
4. CD1a was retrieved from DAKO (Glostrup, Denmark) and used with a citrate pretreatment at 1 : 50 dilution.
5. CD117 was purchased from DAKO (Glostrup, Denmark) and used at 1 : 100 dilution without any pretreatment.

For all procedures, 3,3-diaminobenzidinetetrahydrochloride (DAB, Vector laboratories) represented the chromogen, while hematoxylin (Sigma, Italy), the nuclear counterstain. All immunohistochemical stainings were performed by automatic machine according to the labels and indications of the producers.

Cell count was performed in both epithelial layer and lamina propria. In the epithelium, cell count was expressed as the number of positive cells per 100 enterocytes. This evaluation was performed for the whole mucosa and, additionally, selectively in the tip and at the base of each villum. For this last purpose, villi were divided into three thirds so that the count of the tip was performed in the apical third of the villum and the count of the base in the lower third. The count in the tip and base was expressed as the number of positive cells per 100 enterocytes as well. Finally, a ratio between tip and base count was calculated.

In the lamina propria, the count of positive cells was carried out in six and a half high-power fields (400x magnification), equivalent to 1 mm^2^, and it was expressed as the number of positive cells per mm^2^, as reported elsewhere [16]. A microscope grid was used to help the cell count.

### 2.3. Statistical Analysis

Continuous variables have been expressed as mean ± standard deviation, discrete variables as proportion/percentages. Comparison between continuous variables was led by ANOVA test, followed by Bonferroni's post hoc analysis for multiple comparisons. Fisher's exact test or chi-squared test for trend was applied to dichotomic measures if the analysis was carried out between two or three groups. Receiver operating curves (ROC) were drawn to estimate sensitivity and specificity and their 95% confidence interval (CI) of cutoff values when NCGS showed substantial differences against celiac disease and negative controls. Additionally, the area under the curve (AUC) of ROC curves was calculated when necessary. All tests were two sided, and statistical significance was set at *p* < 0.05. The statistical analysis was performed using the statistical software GraphPad Prism version 5.0 for Windows, San Diego, California USA.

## 3. Results

### 3.1. Characteristics of Enrolled Patients

We retrospectively analyzed 20 patients with NCGS, 16 celiac disease patients (positive controls), and 16 healthy subjects (negative controls). Baseline and demographic details are displayed in Table 1. Age was similar in the three groups. Subjects with NCGS and celiac disease were more frequently female. All patients with celiac disease had DQ2 and/or DQ8 HLA haplotype, while this haplotype pattern was seen only in 30% of NCGS. Abdominal pain, diarrhea, weight loss, and weakness were more common in subjects with celiac disease and NCGS than in controls. Two patients with NCGS reported allergy to alimentary antigens other than wheat (one nichel and one nuts) and, at the moment of endoscopy, they both excluded such foods from their diet. However, the prevalence of alimentary allergy in NCGS was similar to celiac disease and negative controls (*p* = 0.19, as showed in Table 1). Headache was reported in 45% of NCGS, a higher proportion than celiac disease and negative controls.

### 3.2. CD3 Mucosal Expression

**Figure 1:**
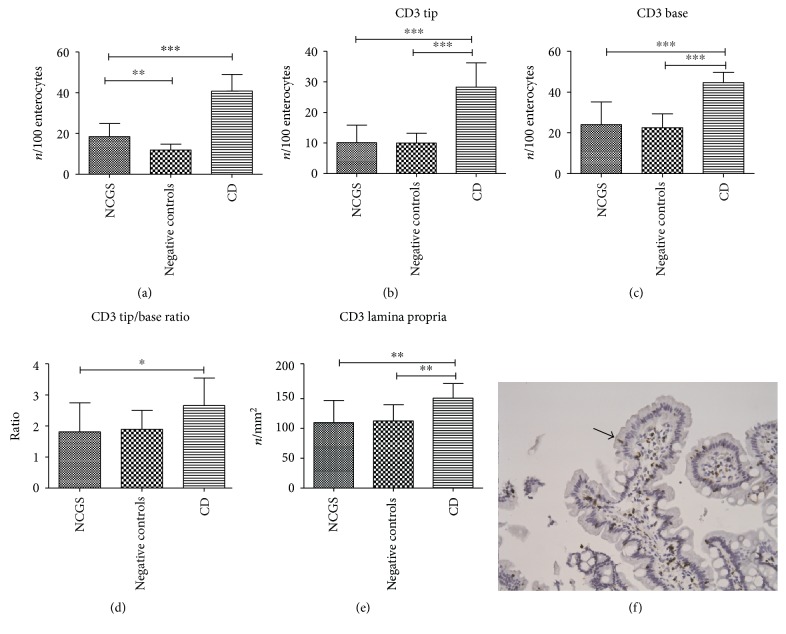
Immunohistochemical expression of CD3 in duodenal mucosa, both epithelial and lamina propria (a–e). Bars and error bars represent mean and standard deviation, respectively. ^∗^*p* < 0.05, ^∗∗^*p* < 0.01, and ^∗∗∗^*p* < 0.001. (f) An immunohistochemical picture of a patient with NCGS: positive cells are stained in brown, magnification ×200. An arrow highlights positive cells.

The total count of CD3 in the epithelial layer showed that patients with NCGS had a density of IELs (18.5 ± 6.4 cells/100 enterocytes) lower than celiac disease (40.8 ± 8.1 cells/100 enterocytes) and higher than negative controls (11.9 ± 2.8 cells/100 enterocytes) (*p* < 0.001), as shown in Figure 1(a). The infiltration of IELs at the villous tip was similar between NCGS and controls (10.4 ± 5.9 and 10.2 ± 3.3 cells/100 enterocytes, resp.), but lower than celiac disease (29.0 ± 8.1 cells/100 enterocytes, *p* < 0.001) as displayed in Figure 1(b). CD3 count at the base of the villi showed a similar pattern (NCGS and negative controls: 6.0 ± 2.8 and 5.6 ± 1.7 cells/100 enterocytes, resp.; celiac disease: 11.2 ± 1.3 cells/100 enterocytes, *p* < 0.001, Figure 1(c)). Moreover, the tip/base ratio of CD3 in NCGS was lower than celiac disease (1.8 ± 0.9 versus 2.6 ± 0.9, resp., *p* = 0.02), while no difference with negative controls was detected (Figure 1(d)). In the lamina propria, the density of CD3^+^ cells in NCGS (110.0 ± 36.9 cells/mm^2^) was similar to negative controls (112.5 ± 27.2 cells/mm^2^) and inferior to celiac disease (150.7 ± 24.8 cells/mm^2^, *p* = 0.002), as shown in Figure 1(e). In Figure 1(f), a characteristic immunohistochemical picture of a patient with NCGS is reported.

We could not draw ROC curves because all values of sensitivity and specificity were <50%.

### 3.3. CD4 Mucosal Expression

**Figure 2:**
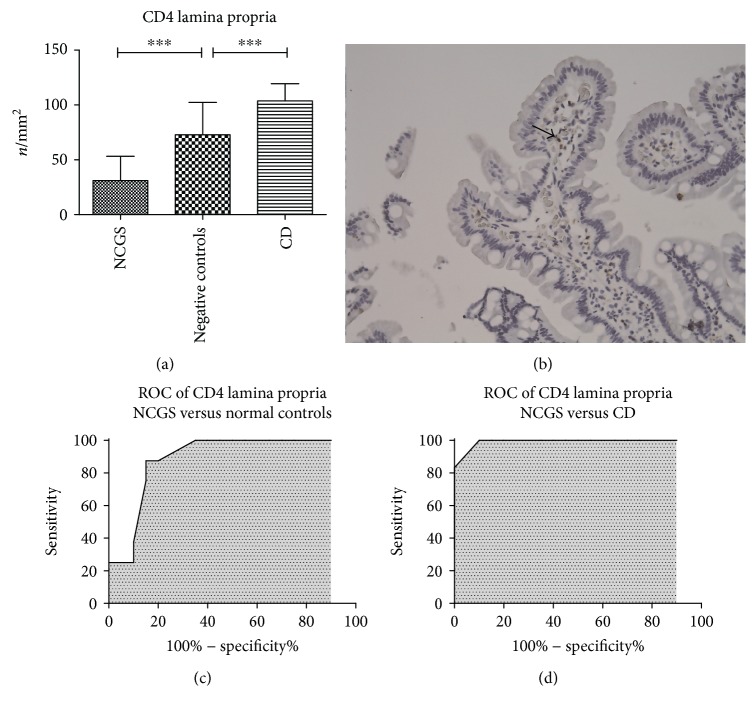
Immunohistochemical expression of CD4 in lamina propria (a). Bars and error bars represent mean and standard deviation, respectively. ^∗∗∗^*p* < 0.001. (b) An immunohistochemical picture of a patient with NCGS: positive cells are stained in brown, magnification ×200. (c and d) ROC curves of sensitivity analysis of CD4 count as a discriminative factor between NCGS and celiac disease or normal controls. An arrow highlights positive cells.

CD4 lymphocytes were only sporadically expressed in the epithelial layer; therefore, a quantitative evaluation was not possible. CD4 was mostly expressed in the lamina propria. We found that patients with celiac disease had a higher density of positive cells than negative controls and NCGS (103.7 ± 15.7, 72.5 ± 29.5, and 31.0 ± 22.2 cells/mm^2^, resp., *p* < 0.001), as reported in Figure 2(a). Therefore, NCGS demonstrated the lowest infiltration of CD4 lymphocytes in the lamina propria. In Figure 2(b), we displayed the immunohistochemical portrait of a case of NCGS.

We draw a ROC curve to differentiate between NCGS and controls. A cutoff of 47.5 cells/mm^2^ showed a sensitivity of 87.5% (95% CI 61.6–98.4%), a specificity of 85% (95% CI 62.1–96.8%), and an AUC of 0.887 (Figure 2(c)). Furthermore, a value of 70 cells/mm^2^ could discriminate between NCGS and celiac disease with a sensitivity of 100% (95% CI 73.5–100%) and a specificity of 90% (95% CI 68.3–98.8%) with an AUC of 0.992 (Figure 2(d)).

### 3.4. CD8 Mucosal Expression

**Figure 3:**
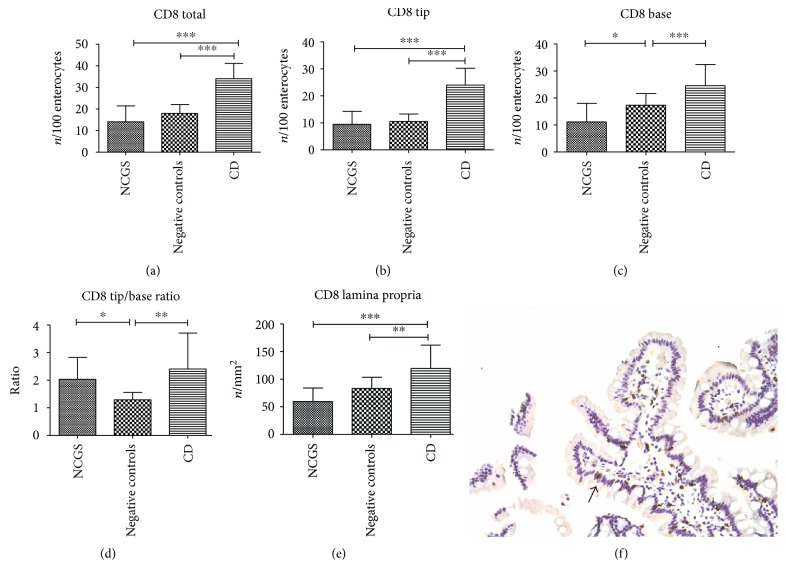
Immunohistochemical expression of CD8 in duodenal mucosa, both epithelial and lamina propria (a–e). Bars and error bars represent mean and standard deviation, respectively. ^∗^*p* < 0.05, ^∗∗^*p* < 0.01, and ^∗∗∗^*p* < 0.001. (f) An immunohistochemical picture of a patient with NCGS: positive cells are stained in brown, magnification ×200. An arrow highlights positive cells.

The overall count of CD8^+^ cells in the epithelial layer demonstrated that NCGS and negative controls had similar levels (14.0 ± 7.4 and 17.9 ± 4.2 cells/100 enterocytes, resp.), lower than patients with celiac disease (34.0 ± 7.1 cells/100 enterocytes, *p* < 0.001). These results are reported in Figure 3(a). The same picture occurred for CD8^+^ cells at villous tip, where celiac disease exhibited a higher density (22.8 ± 5.9 cells/100 enterocytes) than NCGS and negative controls (9.0 ± 4.6 and 10.0 ± 2.6 cells/100 enterocytes, resp., *p* < 0.001) as displayed in Figure 3(b). The expression of CD8 at the base of the villi was the lowest for NCGS (5.1 ± 3.1 cells/100 enterocytes), while, as summarized in Figure 3(c), negative controls and celiac disease were characterized by progressively increasing levels (7.9 ± 1.9 and 11.2 ± 3.6 cells/100 enterocytes, resp., *p* < 0.001). In Figure 3(d), values of tip/base ratio have been reported: in this case, NCGS and celiac disease showed similar values (2.0 ± 0.8 and 2.4 ± 1.3, resp.), statistically higher than negative controls (1.3 ± 0.3, *p* = 0.003). The expression of CD8 in the lamina propria was superior for celiac disease (112.0 ± 39.9 cells/mm^2^) than for NCGS and negative controls (55.9 ± 22.8 and 78.1 ± 18.9 cells/mm^2^, resp., *p* < 0.001, Figure 3(e)). In Figure 3(f) an immunohistochemical picture of a patient with NCGS is shown.

We could not draw ROC curves because values of sensitivity and specificity were <50%.

### 3.5. CD1a and CD117 Mucosal Expression

The immunohistochemical staining for CD1a provided no positivity in any of the enrolled cases (NCGS as well as celiac disease and controls).

**Figure 4:**
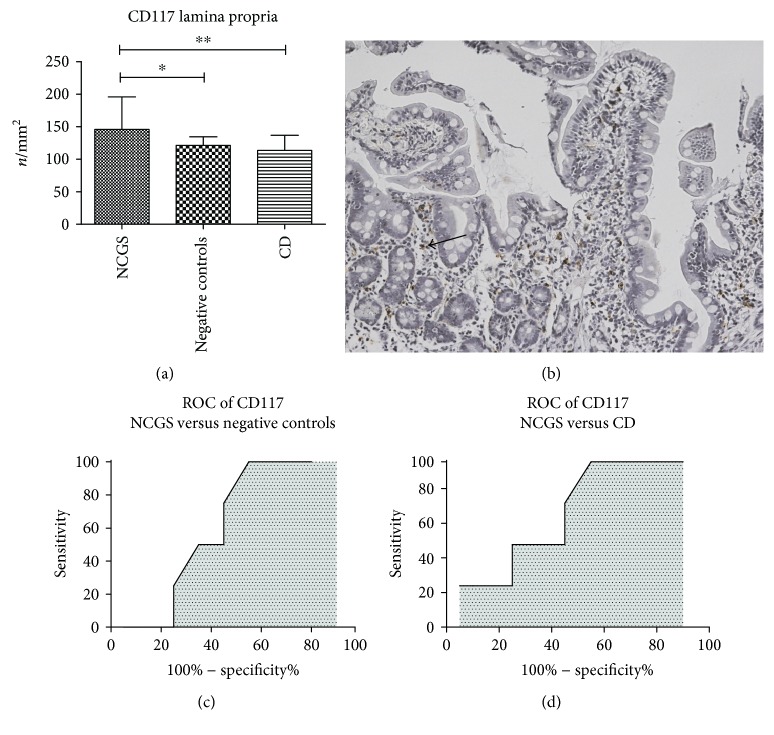
Immunohistochemical expression of CD117 in lamina propria (a). Bars and error bars represent mean and standard deviation, respectively. ^∗^*p* < 0.05 and ^∗∗^*p* < 0.01. (b) An immunohistochemical picture of a patient with NCGS: positive cells are stained in brown, magnification ×200. (c and d) ROC curves of sensitivity analysis of CD117 count as a discriminative factor between NCGS and celiac disease or normal controls. An arrow highlights positive cells.

We did not detect CD117 positivity in the epithelial layer. Therefore, CD117 was expressed only in the lamina propria. In this case, we found that the density of CD117 cells was the highest for patients with NCGS (145.8 ± 49.9 cells/mm^2^, with a *p* = 0.009), while negative controls and celiac disease had lower levels (121.3 ± 13.1 and 113.5 ± 23.4 cells/mm^2^, resp., see Figure 4(a)). We reported an immunohistochemical CD117 picture of a patient with NCGS in Figure 4(b).

However, ROC curve did not allow finding reliable cutoff values for NCGS. A value of 132.5 cells/mm^2^ distinguished between NCGS and negative controls with a sensitivity of 75% (95% CI 56.6–88.5%), a specificity of 55% (95% CI 38.5–70.7%), and an AUC of 0.625 (Figure 4(c)). Similarly, a value of 134 cells/mm^2^ discriminated between NCGS and celiac disease with a sensitivity of 75% (95% CI 53.3–90.2%), a specificity of 55% (95% CI 38.5–70.7) and an AUC of 0.694 (Figure 4(d)).

## 4. Discussion

NCGS is a hot topic in the panorama of gluten-related disorders with well-defined clinical criteria, which have not been supported by specific diagnostic investigations until now. Some early studies aiming to explore its pathogenesis had hypothesized a derangement of innate immunity response to gluten. Starting from this concept, further investigations have been conducted in order to shed a new light on the nature of inflammatory response and microscopic alterations in NCGS. However, these studies have shown conflicting results. For example, Di Sabatino et al. [17] did not demonstrate any difference in the expression of innate cytokines, such as interleukin- (IL-) 15, tumor necrosis factor-alpha, IL-1 beta, IL-6, IL-12p70, IL-23, IL-27, and IL-32 alpha, in comparison to negative controls. Similarly, a short-term gluten challenge in patients with NCGS did not lead to increase in transcript levels of IL-8 and CCL2 chemokines [18]. An *in vitro* experiment demonstrated that gliadin did not induce inflammation in organ culture biopsies from NCGS patients nor activation of peripheral basophils [19]. Regarding the immune-related cell features, some authors reported in NCGS an intermediate infiltration of CD3 IELs between celiac disease and healthy controls [5], while others did not demonstrate any increase in IELs compared to normal subjects [17]. Nevertheless, this last study had the relevant limit of having enrolled patients with self-reported gluten sensitivity. Analogously, a disagreement has been reported for eosinophil mucosal infiltration: while Di Sabatino et al. [17] did not find any difference compared to healthy controls, Carroccio et al. [2] displayed an increase of eosinophils in duodenal and colonic mucosa. Finally, a recent multicenter study confirmed that NCGS is affected by an epithelial damage and an impairment in mucosal barrier similar to celiac disease, corroborated by increased levels of fatty acid-binding protein 2 and enhanced permeability [20, 21]. The same study found further evidence supporting innate immunity activation, that is, the overexpression of sCD14 and lipopolysaccharide-binding protein.

In the present study, we confirmed that patients with NCGS show levels of CD3 IELs higher than healthy controls but lower than celiac disease [5, 9]. Additionally, we found that the tip/base ratio of CD8 IELs in NCGS resembles that of celiac disease. Indeed, in celiac disease, IELs show a peculiar pattern of distribution, with a more enhanced infiltration at the villous tip [22, 23]. This feature could represent a common characteristic of gluten-related disorders.

Another singular finding of our study is the very low expression of CD4 lymphocytes in the lamina propria of patients with NCGS. CD4 staining usually identifies the T helper lymphocytes, whose main function is to start immune response to antigens. In the pathogenesis of celiac disease, gluten peptides are recognized by antigen-presenting cells in the lamina propria, which bind gliadin epitopes to membrane major histocompatibility complex (such as DQ2 and 8 HLA), thus presenting them to T helper lymphocytes [24]. They elicit the immune response to gluten through the activation of B and T cytotoxic lymphocytes. In NCGS, the involvement of DQ2-8 HLA is not peculiar; therefore, a mechanism, which is unbound from antigen-presenting cells and T helper lymphocytes, may be hypothesized. At this regard, some evidences seem to support this point of view. Indeed, it has been shown that the gliadin fragment p31-43 may induce a proinflammatory shift through the activation of MyD88, an adaptor of TLRs, and both MyD88 and TLRs have been implied in the pathogenesis of NCGS [5, 25, 26]. Moreover, some gliadin peptides can directly activate peripheral monocytes by binding TLR4, thus inducing NFkB transcription [27]. Finally, the amylase-trypsin inhibitors, which have been recently acknowledged as the trigger of NCGS [28–30], can activate TLR4. All these evidences seem to support an alternative pathogenetic pathway for NCGS, in which gliadin triggers a direct action through TLRs, without the involvement of the classical epitope presentation mediated by antigen-presenting cells and T helper lymphocytes. In this perspective, our findings could be in agreement with this hypothesis.

Additionally, we assessed submucosal pattern of some antigen-presenting cell expression (i.e., Langerhans cells) by CD1a and CD117 immunohistochemical detection. However, we did not find CD1a-positive cells in the lamina propria, a result which may be expected, since Langerhans cells are mainly homed in gut lymph nodes [28]. On the other hand, the count of CD117 cells in the lamina propria demonstrated a higher number of positive cells in NCGS than in celiac disease and normal controls. CD117 is a marker that can also identify subsets other than Langerhans cells, such as mast cells and pacemaker Cajal cells. However, since the cells of Cajal are localized in deep submucosa and muscular layer, in myenteric ganglia, we can conclude that CD117 cells in the lamina propria are essentially mast cells. Interestingly, a role for mast cells in the pathogenesis of gluten-related disorders has been demonstrated. Under stimulation of p31-43, mast cells may release proinflammatory cytokines through a signaling mediated by MyD88 [31]. Moreover, p31-43 is able to bind TLRs on mast cells, thus leading to the secretion of proinflammatory cytokines and to the maturation of B lymphocytes [32]. Therefore, it may be hypothesized that mast cells could be both the pathogenetic and histopathological keystone of NCGS. Indeed, a high rate of allergic sensitivity to food molecules other than wheat has been pointed up in literature for NCGS patients [33]. Of note, the prevalence of food allergy did not vary amongst the three groups; therefore, we can presume that the increase in mast cells in NCGS cannot be attributed to this factor. However, the immunohistochemistry for tryptase is recognized as a more sensitive method to depict mast cells than CD117. The lack of this staining could be a limit for our analysis.

Other limitations of the present study should be the suboptimal sample size and the lack of data aimed to investigate whether a gluten-free diet was able to reverse histological pattern in subjects with NCGS.

In conclusion, the most important findings of our research are the increase of mast cells and decrease of T helper lymphocytes in duodenal lamina propria of NCGS. The count of CD117 did not show a satisfying diagnostic performance (sensitivity of 75% and specificity of 55%), while a low CD4 count achieved more interesting values (sensitivity of 100% and specificity of 90% against celiac disease). Therefore, we believe that the combination of CD4 and CD117, as well as the characterization of IELs could be useful to support a clinical diagnosis of NCGS. The diagnosis of this disease is nowadays based only on clinical features and, for this reason, a simple and nonarbitrary marker of disease is attractive. In this context, further studies on a large number of patients are warranted to give a real support to the findings of this preliminary experience.

## Figures and Tables

**Table 1:** Main demographic and clinical features of patients with nonceliac gluten sensitivity (NCGS), celiac disease (CD), and normal controls. The *χ*^2^ for trend was employed for statistical analysis.

	NCGS (*n* = 20)	CD (*n* = 16)	Negative controls (*n* = 16)	*p* value
Female/male sex	17/3	12/4	8/8	0.02
Age	33.1 ± 14.4	33.2 ± 11.2	28.5 ± 11.1	0.49
DQ2/8 (*n* and %)	6 (30%)	16 (100%)	3 (18.8%)	0.72
Iron deficiency anemia (*n* and %)	4 (20%)	5 (31.2%)	0 (0%)	0.14
Allergy to alimentary antigens other than wheat (*n* and %)	2 (10%)	1 (6.2%)	1 (6.2%)	0.19
Autoimmune thyroiditis (*n* and %)	4 (20%)	4 (25%)	0 (0%)	0.12
Weight loss (*n* and %)	10 (50%)	10 (62.5%)	0 (0%)	0.004
Abdominal pain (*n* and %)	16 (80%)	12 (75%)	6 (37.5%)	0.01
Diarrhea (*n* and %)	11 (55%)	10 (62.5%)	3 (18.8%)	0.04
Weakness (*n* and %)	9 (45%)	5 (31.2%)	1 (6.2%)	0.01
Headache (*n* and %)	9 (45%)	2 (12.5%)	2 (12.5%)	0.02

## References

[1] Fasano A., Sapone A., Zevallo V., Schuppan D. (2015). Nonceliac gluten sensitivity. *Gastroenterology*.

[2] Carroccio A., Mansueto P., Iacono G. (2012). Non-celiac wheat sensitivity diagnosed by double-blind placebo-controlled challenge: exploring anew clinical entity. *The American Journal of Gastroenterology*.

[3] Volta U., De Giorgio R. (2012). New understanding of gluten sensitivity. *Nature Reviews Gastroenterology & Hepatology*.

[4] Caio G., Riegler G., Patturelli M., Facchiano A., De Magistris L., Sapone A. (2017). Pathophysiology of non-celiac gluten sensitivity: where are we now?. *Minerva Gastroenterologica e Dietologica*.

[5] Sapone A., Lammers K. M., Casolaro V. (2011). Divergence of gut permeability and mucosal immune gene expression in two gluten-associated conditions: celiac disease and gluten sensitivity. *BMC Medicine*.

[6] Volta U., Tovoli F., Cicola R. (2012). Serological tests in gluten sensitivity (non celiac gluten intolerance). *Journal of Clinical Gastroenterology*.

[7] Catassi C., Bai J. C., Bonaz B. (2013). Non-celiac gluten sensitivity: the new frontier of gluten related disorders. *Nutrients*.

[8] Francavilla R., Cristofori F., Castellaneta S. (2014). Clinical, serologic, and histologic features of gluten sensitivity in children. *Journal of Pediatrics*.

[9] Losurdo G., Piscitelli D., Giangaspero A. (2015). Evolution of nonspecific duodenal lymphocytosis over 2 years of follow-up. *World Journal of Gastroenterology*.

[10] Rostami K., Aldulaimi D., Holmes G. (2015). Microscopic enteritis: Bucharest consensus. *World Journal of Gastroenterology*.

[11] Ierardi E., Losurdo G., Iannone A. (2017). Lymphocytic duodenitis or microscopic enteritis and gluten-related conditions: what needs to be explored?. *Annals of Gastroenterology*.

[12] Villanacci V., Lanzini A., Lanzarotto F., Ricci C. (2013). Observations on the paper of Carroccio et al. “non-celiac wheat sensitivity diagnosed by double-blind placebo-controlled challenge: exploring a new clinical entity”. *The American Journal of Gastroenterology*.

[13] Catassi C., Elli L., Bonaz B. (2015). Diagnosis of non-celiac gluten sensitivity (NCGS): the Salerno experts’ criteria. *Nutrients*.

[14] Ludvigsson J. F., Bai J. C., Biagi F., British Society of Gastroenterology (2014). Diagnosis and management of adult coeliac disease: guidelines from the British Society of Gastroenterology. *Gut*.

[15] Moore H. M., Kelly A. B., Jewell S. D. (2011). Biospecimen reporting for improved study quality (BRISQ). *Cancer Cytopathology*.

[16] Tosco A., Maglio M., Paparo F., Greco L., Troncone R., Auricchio R. (2015). Discriminant score for celiac disease based on immunohistochemical analysis of duodenal biopsies. *Journal of Pediatric Gastroenterology and Nutrition*.

[17] Di Sabatino A., Giuffrida P., Fornasa G. (2016). Innate and adaptive immunity in self-reported nonceliac gluten sensitivity *versus* celiac disease. *Digestive and Liver Disease*.

[18] Brottveit M., Beitnes A. C., Tollefsen S. (2013). Mucosal cytokine response after short-term gluten challenge in celiac disease and non-celiac gluten sensitivity. *The American Journal of Gastroenterology*.

[19] Bucci C., Zingone F., Russo I. (2013). Gliadin does not induce mucosal inflammation or basophil activation in patients with nonceliac gluten sensitivity. *Clinical Gastroenterology and Hepatology*.

[20] Uhde M., Ajamian M., Caio G. (2016). Intestinal cell damage and systemic immune activation in individuals reporting sensitivity to wheat in the absence of coeliac disease. *Gut*.

[21] Hollon J., Puppa E. L., Greenwald B., Goldberg E., Guerrerio A., Fasano A. (2015). Effect of gliadin on permeability of intestinal biopsy explants from celiac disease patients and patients with non-celiac gluten sensitivity. *Nutrients*.

[22] Mino M., Lauwers G. Y. (2003). Role of lymphocytic immunophenotyping in the diagnosis of gluten-sensitive enteropathy with preserved villous architecture. *The American Journal of Surgical Pathology*.

[23] Biagi F., Luinetti O., Campanella J. (2004). Intraepithelial lymphocytes in the villous tip: do they indicate potential coeliac disease?. *Journal of Clinical Pathology*.

[24] Branski D., Fasano A., Troncone R. (2006). Latest developments in the pathogenesis and treatment of celiac disease. *The Journal of Pediatrics*.

[25] Araya R. E., Gomez Castro M. F., Carasi P. (2016). Mechanisms of innate immune activation by gluten peptide p31-43 in mice. *American Journal of Physiology. Gastrointestinal and Liver Physiology*.

[26] Losurdo G., Giorgio F., Piscitelli D. (2016). May the assessment of baseline mucosal molecular pattern predict the development of gluten related disorders among microscopic enteritis?. *World Journal of Gastroenterology*.

[27] Palová-Jelínková L., Dáňová K., Drašarová H. (2013). Pepsin digest of wheat gliadin fraction increases production of IL-1*β* via TLR4/MyD88/TRIF/MAPK/NF-*κ*B signaling pathway and an NLRP3 inflammasome activation. *PLoS One*.

[28] Zevallos V. F., Raker V., Tenzer S. (2017). Nutritional wheat amylase-trypsin inhibitors promote intestinal inflammation via activation of myeloid cells. *Gastroenterology*.

[29] Junker Y., Zeissig S., Kim S. J. (2012). Wheat amylase trypsin inhibitors drive intestinal inflammation via activation of toll-like receptor 4. *The Journal of Experimental Medicine*.

[30] Schuppan D., Zevallos V. (2015). Wheat amylase trypsin inhibitors as nutritional activators of innate immunity. *Digestive Diseases*.

[31] Frossi B., Tripodo C., Guarnotta C. (2017). Mast cells are associated with the onset and progression of celiac disease. *Journal of Allergy and Clinical Immunology*.

[32] Merluzzi S., Frossi B., Gri G., Parusso S., Tripodo C., Pucillo C. (2010). Mast cells enhance proliferation of B lymphocytes and drive their differentiation toward IgA-secreting plasma cells. *Blood*.

[33] Carroccio A., Mansueto P., D'Alcamo A., Iacono G. (2013). Non-celiac wheat sensitivity as an allergic condition: personal experience and narrative review. *The American Journal of Gastroenterology*.

